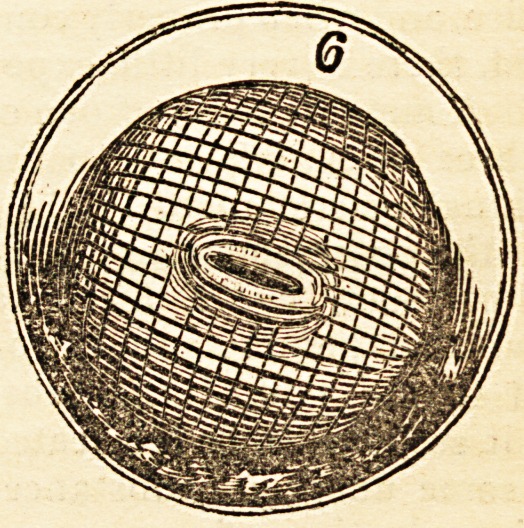# Extra-Limites

**Published:** 1840-04-01

**Authors:** 


### EXTRA-LIMITES.

### CITY OF LIMERICK INFIRMARY.

The two following cases of Compound Fracture of the Skull may prove interesting to some of your readers; tending, as one of them does, to shew the amount of injury the brain and its coverings may sustain, without any permanent bad consequences in the young subject, when compared with the result of similar injuries in the adult. It may appear to some, that if the operation of trephining and raising the depressed bone had not been resorted to at so early a period in Lynch's case, the result would have been different. However, I conceive that the rule of practice adopted in the case, (and which is generally adopted in this part of the country, where injuries of the head are accidents of very frequent occurrence) was the most advisable; namely, not to wait for the supervention of bad symptoms in cases of compound depressed fracture occurring in adults—where the depression is so considerable that its presence must cause inflammation of the dura mater, or of the brain itself—but to raise the bone as soon as possible. At the same time, in cases where the depression is not so great, although the symptoms of compression may be urgent, I believe the best practice is to try if such symptoms may not be caused by the effusion of blood on the dura mater, and not to interfere until the effect of other treatment has been tried.

It is no easy matter to define the amount of depression of the bones of the skull, which would lead the practitioner in one case to elevate it, and in the other to wait for symptoms to guide his treatment; but I would say, that the extent of depressed bone would not influence me so much as the depth to which it is forced on the brain; and that, generally speaking, in adults where the bone is so much depressed beneath the level of the uninjured bone that an elevator or some such blunt instrument could be easily introduced between them, I would at once elevate without waiting for symptoms of compression, should such not be present at the time.

Such was the treatment adopted in Lynch's case, and although the result was unfavourable; yet I conceive it afforded the best chance of success, as until the bone was elevated I was unaware that the dura mater was torn, and the substance of the brain injured—and he had not at any period symptoms of inflammation of the brain or its coverings.

In the young subject, however, I have seen several instances of very extensive depresssion of the bones of the skull, where recovery took place without any surgical interference; the bone gradually assuming nearly its natural level according as the cranium expanded.

### Compound Fracture of the Head.

Thomas Lynch, 24 years of age, was admitted October 2nd, 1838; the previous night he received a blow of a watchman's pole, (which had an iron ferule on its end) on the left side of the head, which knocked him down, inflicting a lacerated wound of the scalp, and a depressed fracture of the left parietal bone—the wound bled rather profusely, but he quickly recovered the shock, and was not brought to hospital until late the following day. A consultation was soon held, and the bone being much depressed, the unanimous opinion of the other surgeons of the hospital was that the bone should be elevated without delay, although at this period no urgent symptoms existed. I found it necessary to remove with the trephine about three-quarters of a circle of bone to enable me to raise the depressed pieces which were forced through the dura mater, &c.—the lips of the wound were brought together by suture—evaporating lotions ordered to the entire head, and aperients given. He slept well the two following nights—the pulse 62. Bowels regular.

October 6th. Makes no complaint of pain of the head; his pulse is less frequent the last day or two, only 46. He was ordered calomel and antimonial powder, and a blister to the nape of the neck—the wound appears to be united.

October 9th. Pulse 50. Some purulent discharge from wound—sleeps well, tongue clean, complains of numbness of his right hand.

Oct. 11. Pulse 50. The wound seems raised, as if by a fungus, and about to open where united. Mouth not sore.

12th. Lynch was suddenly attacked this morning by a fit of insensibility, and became incoherent—his tongue is partly paralysed—the fungus is not much more raised—during the two following days he continued nearly insensible, his extremities cold, and the right side of his body became entirely paralysed—the fungus cerebri burst through the wound and rapidly enlarged—he then recovered his consciousness in a great measure.

Oct. 20th. The fungus, which at this period was the size of a small orange, sloughed away to the level of the bone—he is perfectly sensible, but cannot articulate—his appetite is very great—he does not wait to be fed, when he sees food, but seizes it with the hand he can command.

26th. He became again insensible this morning—appeared to be on the point of dying—he however quickly rallied.

29th. The cornea of the right eye has gradually become opaque, within the last few days—a bad sore has formed over the sacrum.

November 9th. The external ear of the same side has been attacked by gangrenous ulceration, which quickly destroyed it—the eye has become staphylomatous—he eats and drinks with great voracity, and knows every attendant—the fungus cerebri re-appeared once or twice, and sloughed—the discharge from the head has been profuse—he however lingered until the night of the 14th November, when he died. No opportunity was had of making a post-mortem examination.

### COMPOUND FRACTURE OF THE SKULL.

Patrick Farrell, aged seven years, was admitted 17th January, 1839. Two days before he received a kick from a horse on the forehead, which caused a lacerated wound over the left eye, and an extensive fracture of the frontal bone, with considerable depression—he complained of pain in the wound—his tongue was white, pulse quick—but had no urgent symptom. The head was directed to be shaved, and evaporating lotions ordered.

20th. Going on well—a small quantity of what appeared to be dura mater was removed from between the fractured bones—at each dressing, for some days after, some of the brain came away through the same opening, his general health, however, continued tolerably good—granulations began to shoot up from the depressed bone, and the surrounding soft parts—the wound gradually closed in.

February 15th. A piece of dead bone was this day removed, and subsequently three or four large pieces were taken from under the loose granulations which filled the wound. The pulsation of the brain now became evident—the boy was discharged well on the 25th: altogether about the size of a half-crown piece of both tables of the skull was detached during his stay in the hospital.

### Case of Aneurism by Anastomosis.

Thomas Kenny, 19 years old, of sickly appearance, presented himself with an ulcer over the second phalanx of the second toe of his right foot; the ulcer was about the size of a shilling, covered with loose, fungous granulations not very painful, the surrounding integument was of a purplish colour; it bled freely on the slightest touch or attempt to walk; a probe passed through the granulations easily reached the bones of the toe which were evidently diseased. Under the same toe a tumour nearly as large as a hen's egg appeared, which extended from the point of the toe to the articulation of the phalanges and metatarsal bones; it had much the appearance of an abscess, and had previously to his becoming my patient been punctured by a lancet; but the rapid flow of arterial blood rendered it necessary to close the puncture quickly. The patient's attention was not drawn to the tumour until after the appearance of a small swelling on the dorsum of the toe, which he conceived to be a corn and scraped with a knife; this never healed, but gradually enlarged during the last two months, which obliged him to seek relief. The tumour was partially compressible, nearly of the colour of the skin, conveyed a peculiar vibratory pulsation to the finger, synchronous with the pulse; the anterior tibial artery on the dorsum of the foot had a similar feel, and pulsated more strongly than usual, as did the peroneal also; pressure on those arteries partially diminished the pulsation in tumour. In consultation it was decided to remove the toe at its articulation with the metatarsus, together with the tumour as far as practicable.

Accordingly, on the 6th April, 1836, I made rapidly two incisions which met at the articulation, which fortunately was immediately opened, and the toe and tumour removed. Ten or twelve arteries bled smartly—they were secured and the flaps of the wound brought together, and cold applications used.

April 9th—there was some oozing of blood, which was easily arrested by cold; it however returned the following night, and became so profuse as to oblige me to remove the dressings; four or five arteries were bleeding from the flap next the great toe. I found it impossible to draw them out and tie them separately, and therefore introduced an armed needle into the angle of the wound, and included in the ligature that flap with the vessels. From this time there was no return of hæmorrhage; the wound granulated kindly, and the patient's general health improved.

May 7th. The report states, that a small swelling has appeared near the upper angle of the wound, at the side near the third toe, which pulsates feebly; the wound cicatrized, and he was discharged well on the 23d May. This young man called on me about two years after the operation; he lived in an adjoining county—enjoyed excellent health, and has had no return of the disease.

### Case of Gangrene of the Lung.

Michael Sullivan, a strong muscular man, but of intemperate habits, was admitted April 10th, 1838. He stated that, nine days before (while in a state of intoxication) he was severely beaten in the County Kerry, and that on the following day he was attacked with cough and pain of chest, for which he was blooded, with temporary relief. It would appear that no further treatment was adopted until he was brought to hospital, having become much worse in the interval. On admission he complained of very severe pain of his right side and cough; the expectoration was scanty and dark coloured, occasionally streaked with blood, and the fetor was peculiarly disagreeable; the respiration was quick and oppressed—pulse feeble and rapid, his countenance pale and swollen; he lay on his back and breathed loudly through his nose. The entire of the right side of his chest sounded dull on percussion, respiration inaudible—the left lung appeared healthy. Two or three trifling lacerated wounds were visible on his face, and his lower lip was divided—there was also a contusion over the second and third ribs on the right side. The feeling of suffocation and debility increased rapidly, and he died early on the morning of the 17th April. On examining the chest, the lung appeared to be nearly in all parts adherent to the pectoral parietes by means of recently effused lymph. On raising the upper ribs, a gangrenous cavity of the size of a half-crown-piece was opened accidentally, owing to the close attachment of one of its walls to the rib—its cavity was filled with a dark greenish fluid of extreme fetor—it was circumscribed, and corresponded to the mark of contusion on the skin covering the ribs, which were unbroken—there was a similar cavity, but of much greater size, on the surface of the lung lower down, which communicated with some of the bronchial tubes; its contents were similar to that of the other—the remainder of the lung was of a fleshy consistence, of a dark purplish colour—it did not crepitate on pressure; the left lung presented a tolerably healthy appearance, the heart and the abdominal viscera were in a perfectly normal state.*

---

### CASE OF GUN-SHOT WOUND OF ARM, FOLLOWED BY GANGRENE. AMPUTATION AT THE SHOULDER JOINT—SECONDARY HÆMORRHAGE—SUCCESSFUL LIGATURE OF THE SUBCLAVIAN ARTERY.

THE following case has been communicated to us by Mr. W. W. Stafford of Hailsham, the gentleman under whose care it occurred, and to whose credit it redounds. We have much pleasure in inserting it.

July 25th, Abraham Prodger, aged 17, a healthy lad of a robust constitution, who had lived sparingly for some months past, was incautiously drawing a gun from a hedge, in which he had previously placed it, when a bough caught the trigger and discharged the contents, consisting of several pieces of cut lead, through the right arm, entering at the inner edge of the biceps muscle about two and a half inches below the axilla and neck—in its escape at the posterior edge of the deltoid within an inch and a half of the acromion process, having passed at the inner side of the humerus. The bone was not injured—the hæmorrhage considerable. A friend of mine, Mr. Holman, happened to be near the place at the time of the accident, and immediately placed a compress on the artery above the wound, and a simple roller tightly round the arm from the shoulder downwards to near the elbow—sensation in the hand and arm was perfect, but no pulsation of the artery could be felt at the wrist. The degree of collapse was not very considerable, and re-action took place in the space of four hours after the accident, unattended by hæmorrhage. About this time I saw him, the arm and hand slightly swollen and rather inclined to be cold—gave him a little brandy and water, an anodyne draught, and ordered the arm to have warm flannels constantly applied, but this was not strictly attended to. 26th. Has passed a tolerable night—pain and fever slight—bowels costive—sensation complete—limb warm—but no pulsation could be felt—gave him an aperient, and ordered a continuation of the warm flannels. 27th. Rather agitated from the

* Since putting this Article to press, we have discovered the omission of the name of the Surgeon to the Infirmary, who treated and has communicated the preceding cases. The Letter containing it has been unfortunately mislaid.

fear of an operation being required—countenance somewhat anxious—tongue white and moist—pulse quick, full and incompressible—slight heat and dryness of skin. The limb discolored and cold fingers, livid and stiffened—the cuticle desquamating at several places on the arm—sensation gone—skin tense and hard—crepitation on pressure. I cut the bandages, and a quantity of air escaped from the wound—fætid discharge from the orifice—opium, camphor and ammonia, given every three hours, also wine and good broth. 28th. Tongue white and moist—pulse quick, more feeble, and rather compressible—countenance anxious—bowels confined—skin hot and dry. The gangrene appears to be spreading rapidly. Shoulder considerably swollen—the tumefaction is greatest on the inner side of the deltoid, where there is great discoloration and crepitation, extending above the clavicle, but neither the deltoid or pectoral muscles appear to be implicated, merely the cellular tissue—stale beer grounds poultice, &c. applied to the whole of the shoulder—the opium, &c. also wine and broth, continued *constantly*. 29th. Countenance anxious—pulse quick, feeble and easily compressed—tongue white and moist—dryness and increased heat of skin—bowels costive—passed a tolerable night. A line of demarkation is seen extending round the arm just above the wound—the discoloration above the wound not more extensive than yesterday, and less distinct. Gentle aperient, rep. pilul. &c.—wine every hour.

30th. Countenance much more cheerful—face rather flushed—tongue thinly coated with a brownish colored mucus—pulse small, quick, and not easily compressed—slept at intervals during the night—skin rather hot. The discoloration above the wound almost disappeared—crepitation diminished—swelling round the joint decreased. Rep. pilul. wine and broth.

31st. No discoloration can now be perceived above the wound—swelling diminished—pulse much as yesterday—skin comfortable—tongue moist and brownish at the base—bowels rather confined—countenance free from anxiety. Having the line of demarkation complete, discoloration and crepitation above the wound gone. I determined on the operation of amputation at the shoulder-joint, which, in the presence of my medical friends, I performed in the following manner. A semilunar incision was made on the external part of the shoulder, including nearly the whole of the *deltoid*, and terminating just above the insertion of that muscle. The flap was turned up, leaving the upper part of the humerus and part of the acromion bare. An assistant now rotated the arm, and the head of the bone being felt, the capsular ligament was divided ; the head of the bone was then raised from the glenoid cavity by depressing the extremity of the humerus. The operation was then completed by cutting round the articulatory surface of the head of the bone, and dividing the remaining ligament, muscles, and integuments, terminating the incision in the axilla. From six to eight ounces of blood were lost during the operation ; the arteries being secured and the parts brought together by sutures and adhesive plaster, a pad of lint, several times folded, was placed on the flap, and steady pressure made by the hand, but hæmorrhage having recurred to about three or four ounces, the dressing and sutures were removed, and the surface of the wound again exposed; several coagula were removed, a small artery was then tied, and the wound kept constantly sponged with cold water. As soon as the bleeding had entirely ceased, the wound was dressed as before, and steady pressure was made by the hand for seven or eight hours after. No bleeding occurred—some wine was administered during the operation, and a few minims of sal volatile. Three glasses of wine were given afterwards during the day, also mutton broth, &c. &c. The countenance was rather blanched, and the pulse feeble. The boy slept comfortably after the operation—re-action took place in the space of nine hours. Clammy sweats during sleep. Pulv. opii gr. i. h. s. s.

August 1st. Slept tolerably during the night—tongue moist, and of a brownish coloured base, countenance rather blanched, pulse quick and rather fuller, skin tolerably comfortable—smart pricking pains are felt in the shoulder—bowels confined. Evening. He became very restless and irritable, skin parched and hot—cold sponging from head to foot was used, this was shortly succeeded by a sound and refreshing sleep—diet the same as yesterday.

5th. Pulse somewhat sharp, not so easily compressed, tongue coated, bowels open, pain in the stump increased, a pretty healthy discharge of pus from the wound. Passed a good night.

6th. General health good—discharge increased—some of the dressings removed, and fresh ones applied, as they were becoming fætid—adhesion had taken place to a considerable extent, and the wound looked exceedingly healthy —diet, meat, wine, beer, &c. &c.

12th. About midnight complained of severe throbbing pains in the shoulder, and sensation of great tension, which symptoms were succeeded by a flow of blood mixed with pus and a large quantity of serum. The subclavian was immediately compressed, and the hæmorrhage very shortly after subsided—it was not deemed necessary to compress the artery but for a very short time after the cessation of the bleeding—the loss of blood was inconsiderable—on removing the dressings, the wound appeared very healthy, and its edges were tolerably firmly united by the first intention and by granulations. General health good, countenance not blanched, pulse a little diminished in force—omit the wine.

13th. No recurrence of hæmorrhage, granulations paler, a very small quantity of a sanious discharge exudes by the side of the ligatures at the upper part of the wound, discharge of pus rather diminished, but healthy; general health good, but he feels the loss of his wine. Slept well. 14th. Much the same; resume the wine with a more generous diet. Applied fresh strapping to the wound. 16th. General health good—very cheerful, and has more power. Granulations have regained their redness—the wound cicatrizing—discharge not copious, and seems to procced almost entirely from the surface. The flap feels firm, as if perfectly adherent. 17th. Continued doing well till towards evening, when a throbbing pain took place, accompanied by a sensation of heat and tension. About eight o'clock, slight hæmorrhage occurred from the lower part of the wound to the extent of ℥ss. of arterial blood, which speedily coagulated—the boy much agitated, pulse quick, but not feeble, bowels confined—the wound sponged with cold water; an aperient administered. 18th. No sleep during the night, throbbing pains continued till six o'clock a.m. when a slight discharge of blood, ℥ss. mixed with pus, took place—this relieved the pain. Pulse quick and compressible, skin rather hot and dry, tongue slightly coated, bowels confined. Pil. rhei comp. gr. x. h. s. s. 19th. Passed a good night, free from pain, discharge of pus free and healthy, bowels open, pulse quiet, seems much more composed—the dressings were removed, and the wound presented a healthy appearance, and rapidly cicatrizing—the granulations at the lower part have risen considerably above the surface, and are rather spongy. A small spot of coagulated blood is observed at the apex of one of the granulations, which led me to suppose the hæmorrhage had arisen there. Applied the argent. nit. and pressure by strapping and simple bandage—the ligatures remain firm. 20th. Doing well. 21st. At one a.m. complained of heat and throbbing pain at the shoulder, which was shortly succeeded by a sudden and full stream of blood flowing by the side of the ligatures to an alarming extent—pressure on the subclavian was made by the boy's mother as soon as possible. I found him in a state of extreme collapse, the extremities cold, countenance and lips blanched, respiration hurried—delusions at intervals—" singing hymns, endeavouring to frighten crows by bawling loudly"—pulse very weak, quick, scarcely perceptible—a dose of opium and a little wine, bottles of hot water to the feet, and constant pressure on the subclavian, from 20 to 25 ounces were lost—towards the afternoon re-action took place, accompanied with very slight hæmorrhage, owing to the compress having been a little slackened—this was immediately restrained by pressure on the subclavian artery. I then cut down on the artery above the clavicle, and secured it—very little blood was lost during the operation. The boy was very much exhausted—a glass of wine, and pills with opium, camph. and am. sesq. carb. were given statim—there is a discharge of healthy pus, and the wound looks exceedingly well. 22nd. Slept well during the night—about eight a.m. a small quantity of blood issued from the stump, suppose ℥ij. but was soon stopped by applying cold water—the shoulder was quite warm, spirits good, and countenance more natural than yesterday—pulse small, quick and feeble, tongue white, great thirst. Evening. Skin dry, retching on attempting to swallow any thing, with slight hiccough—air is expelled from the stomach at the same time in large quantities, has kept nothing on the stomach but a few spoonsful of milk—pulse very quick and sharp, bowels costive, pulv. opii h. s. and mustard poultice to the pit of the stomach till it produced a tingling. 23rd. Passed a pretty good night, and retained a cupfull of milk on the stomach—had very little retching or flatulency, pulse 100 and sharper, skin comfortable, countenance without anxiety, tongue white, slight aperient given, and potass. citratis occasionally. After the bowels were relieved, pills with opium, camph. and am. were administered. Evening. Pulse and tongue much the same as in the morning. No retching or hiccough—has taken two teaspoonsful of wine and a few spoonsful of milk and isinglass jelly since morning, repeat the pills, h. s. 24th. Passed a good night. Tongue the same as yesterday, pulse not so sharp, no sickness or hiccough, throbbing of the carotids. Retains his food, bowels confined, skin comfortable, and countenance free from anxiety, stump considerably reduced, one ligature away, granulations sunk below the surface and pale, with a sanious discharge by the side of the ligatures. Lotio nig. pressure by bandages round the stump—jellies, broth, and small quantities of wine and beer. 25th. Slept well, tongue white and moist, pulse not so quick or sharp; circulation much more tranquil, carotids quiet, countenance rather anxious, disposition to sloughing in the stump, and a sanious fœtid discharge. Appetite impaired, less flatulence, retains his food, skin moist, thirst much abated, complains of cramp about the left arm and leg. Bowels confined—lotio acid. nit. to the stump, pil. rhei comp. gr. x. statim sum.—ext. gent., am. carbon. gr. v. ter die cap. Food and wine continued as yesterday, but did not take so much.

26th. Tongue clean and moist, anxiety of countenance, sickness and flatulence very much diminished, bowels once open, slept well, and expresses himself much better. Appetite improved, pulse not so quick as yesterday. Granulations beginning to rise, and are much more vascular—a discharge of healthy pus from the stump—a little fœtid discharge from the neck. Evening. Pulse quicker and sharper, no fever, a little throbbing of the carotids—granulations evidently improving—has taken meat, beer, and brandy and water, retains them without sickness or any unpleasant symptoms. 27th. Slept very well—tongue clean and moist, pulse quick, feeble, and less sharp—bowels confined—appetite improved, countenance tolerably cheerful—feels stronger—a discharge of healthy pus from the stump, granulations vascular and increasing, wound healing, a small sinus in the direction of the ligatures is formed. The remaining ligatures of the stump away, and the boy complained of a pricking sensation in the fingers when they were pulled—the wound above the clavicle was dressed, and the greater part has healed by first intention—a slight healthy puriform discharge escapes from each end.

The ligature of the subclavian came away on the 2nd of September, after which both wounds rapidly healed, and in the course of a fortnight the boy was able to sit up in a chair, and in another week came down stairs.

### CASE OF RUPTURE OF THE AORTA, WITHOUT ANEURISM. By *Edward H. Weale*, M.R.C.S.L. Assistant Surgeon Royal Naval Hospital, Plymouth.

CASES of rupture of the aorta, without aneurism, are of such unfrequent occurrence, more especially when not attributable to external violence, that I am induced to offer for insertion in your Journal, the particulars of a remarkable case, which I had an opportunity of examining *post mortem*, a few days since, in conjunction with my friend Mr. Stewart, of this town, who was the medical attendant of the patient.

Some points in the early history of this case are deserving of notice, and shall be related as briefly as possible. The account of the symptoms precursory to death is nearly verbatim from the recital of Mr. Stewart.

Mr. C———, about 60 years of age, of a very full habit and who had passed his life in an active employment, and in the enjoyment of excellent health, whilst walking some distance from home about a year since, was attacked with very severe pain in the calf of the left leg, which prevented him from returning to his house without help. Mr. Stewart was requested to see him, and found the part tumid, but not inflamed. The tongue soon became greatly swollen, the fauces red, and articulation difficult and much impaired. Supposing that these complaints were symptomatic of some cerebral affection, active means were directed for its relief; and with such success, as to enable him, after a while, to resume his usual occupations, but difficulty of speech, and partial loss of power of the left extremities were permanent. He had lately suffered some severe reverses and consequent depression of spirits. On the 21st of November, about 5 p.m. Mr. S. was sent for; on his arrival, he learnt from Mr. C. who lived a little way in the country, that he had been seized with violent pain in the knee, about 12 o'clock, whilst he was in town, which was so great that he was unable to return home without the assistance of his son; and on reaching his house, while lifting his hand to raise the latch of the door, he experienced "a violent snap," succeeded by great pain between the scapulæ, and a sensation of oppression and fulness. The pain increased in intensity until Mr. S. saw him, when he found him labouring under great restlessness; indeed, so much so that he could scarcely remain still while his pulse was being felt, which was labouring and unequal in volume, but not in frequency. He could take a full inspiration, which increased the pain between the shoulders, and he expressed himself as enormously distended with flatus. An aperient was prescribed, and he promised to call again at ten o'clock, at which time Mr. C. was in bed, and had had a few minutes sleep whilst lying on his back, but the restlessness continued. He was tossing about and said that he could find no relief in any posture, and appeared just the same in every respect as at the first visit. That some large vessel in the thorax had given way Mr. S. did not entertain a doubt, and on calling next morning, he was informed that his patient expired "after two or three gasps," soon after he had left him.

*Sectio Cadaveris.*—The body was remarkably fat, and the face was of a waxy exsanguined aspect. The veins of the neck and anterior part of the thorax, presented the varicose condition, so often attendant on cardiac disease. On section the pericardium was found to contain nearly a quart of semi-coagulated blood, which had escaped from a lacerated opening, about three quarters of an inch in length, on the right and rather posterior part of that portion of the aorta which is covered by the pericardium, and where it emerges from behind the pulmonary artery. The serous membrane surrounding the origins of the great vessels—particularly posteriorly, presented a livid ecchymosed appearance, from the effusion of blood into the subjacent cellular tissue, to such an extent as to give to the aorta a sacculated form, which at first led to the idea that there was an aneurism of that vessel. The heart and its great vessels were carefully removed, for the purpose of a more accurate examination. On introducing the finger into the laceration, it did not find its way into the cylinder of the artery, but merely moved in a sac (containing recent clots) formed by the separation of the external and middle coats. The source of the hæmorrhage was not discovered until the artery was laid open from the left ventricle, when a small transverse opening, barely large enough to admit a quill, was observed immediately behind a semilunar valve, and close to the mouth of the posterior coronary artery. At this spot there was not the slightest indication of ulceration nor erosion, but simply a rent in a part of the vessel, which is naturally thinner than other portions of the trunk. Through this fissure the blood must have escaped, and dissecting off the more elastic external coat to such an extent as to produce the appearances before described, at length ruptured that tunic and filled the pericardium. The calibre of the artery was increased, and the lining membrane was here and there raised from the middle coat by atheromatous matter, mixed with gritty particles. It was so soft as to be scraped off without difficulty by the finger nail. The fibrous tissue had lost its elasticity, and could be torn by the most moderate force. The arteria innominata, left carotid and subclavian were very large, but not apparently diseased. The left ventricle was enormously hypertrophied; its wall being nearly two inches in thickness. The valvular apparatus was healthy. The lungs and abdominal viscera were quite normal.

It is probable that the rupturing of the internal coats of the artery took place when the patient felt the "violent snap," and that the external one was lacerated at some short period before death. The pain in the leg is a singular feature in this case. But in a case of extensive abscess of the right anterior lobe of the brain, which I examined a short time since, the convulsions which occurred frequently during the patient's illness, were usually ushered in by a sensation of numbness and pain in the calf of the left leg, extending up the side.

The inconveniences generally attendant on cadaveric inspections in cases occurring in private practice, and the unwillingness on the part of relatives to have such pursued with that strictness essential to advances in pathology, must, in many instances, prevent medical men from arriving at important truths. In the present instance we were not able to examine the head, although the patient had suffered from the paralytic affection before mentioned.

In some cases of apoplexy which have occurred to my observation within a few years, the arch of the aorta has in general been found deprived of its normal elasticity, sacculated, and the seat of atheromatous and sometimes bony deposits; while the coats of the vessels of the brain, (where we were able to trace the hæmorrhage to its source) were loaded with gritty particles of a similar nature to the matter in the aorta. That sudden death, from the bursting of a large artery where there is no actual aneurismal dilatation, may occasionally occur, without detection, is far from improbable, and such an event may be attributed to a recurrence of apoplexia cerebri, when arterial disease in some other part of the system may have been the cause. Hypertrophy of the left ventricle is also a frequent concomitant, and though itself perhaps secondary to the arterial disease, produces a fatal result by its powerful reaction on a weakened circulatory system, as was probably the case in the present instance.

---

### MR. REID'S CASES.

Mr. Reid, a highly intelligent surgeon of Glasgow, has forwarded to us the following cases, which we have pleasure in inserting.

I. Case of Purpura.

Alexander Gray, turner, aged 27, married, of good constitution and in comfortable circumstances, consulted me on the evening of the 10th April, 1839, after leaving his usual employment. He complained of severe muscular pains in various parts of body, sore throat, languor, thirst and disinclination for food. Pulse 70. Tongue covered with a thick yellow coat. Bowels constipated. Skin natural. No head-ache. Had a rigor three days previous to application. I ordered him an emetic of tart. antim. in divided doses to be followed early in the morning by a purgative of calomel and pulv. jalapæ comp.—11th April. I visited him at mid-day, when, in addition to the symptoms noted yesterday, my attention was directed to the gums, which were swollen, of a dark colour and painful; to the breath, which was very fætid, and to an eruption of petechiæ over both tibiæ. The spots were of a bright red colour, not larger than split-peas—existing in clusters but not confluent, and having no perceptible elevation of cuticle. He likewise complains of pain in knee and elbow-joints, and is very restless. Pulse 90. As the purgative did not act freely, he was ordered a sol. of sulph. magnes. to be repeated towards evening. 12th April. During the night he had two scanty watery stools, which were very dark and emitted a most disagreeable odour. Is compelled from the severe pain of the spine and joints to lie fixed in one posture on his back—has had no sleep during the two preceding nights—is very anxious and alarmed at his present condition and complains of præcordial oppression. The knee and ankle of right leg are considerably enlarged and painful on pressure, and the corresponding foot is œdematous. Pulse 120 strong. I took 24 ounces of blood from his arm and prescribed vin. colchici in large doses, in order to induce purging as well as its anti-rheumatic effect. 13th. The blood drawn yesterday has a very thick buffy coat, is cupped and firm. Has been purged severely, the fæces however still retain the same characters. Can turn better in bed and expresses himself as being a little relieved. Pulse is reduced in frequency and strength. A few livid petechiæ have appeared over the sacrum. The colchicum was continued with the addition of a small quantity of tinct. opii. 14th. Mr. Wm. Lyon, one of our hospital surgeons, visited the patient this afternoon, when he exhibited the following appearance. The face was shrunk and sallow—he was wakeful and restless, though from the violent pain of joints he was unable to turn in bed without assistance. Has no difficulty in breathing nor pain on pressing abdomen—the petechiæ over tibia were fading, those on back enlarging—pulse 110, rather weak—tongue white —gums spongy and separating from the incisors—breath still very disagreeable. Calomel and opium was substituted for the former medicines. 15th. The case has within the last ten hours assumed a most alarming aspect. Two large patches of deep ecchymoses have appeared on right cheek, which has become much swollen, painful and hot to the touch, as if occasioned by hard blows. Blood is extravasated beneath the conjunctival covering of left eye-ball, and also under the mucous surface of lower lip towards the angle of the mouth; the gums bleed on the slightest touch, the fauces are of a deep red, and the fætor of the breath is intolerable. The spots on his back are as large as a shilling, and new ones are forming on every part of the body and upper extremities. Has had two stools mixed with blood, and his urine, though transparent on being voided, soon becomes turbid and exhales a fætor as disagreeable as the fæces. Pulse 120 very weak. Rheumatism a little diminished. The calomel and opium, of which he had only taken two pills, were discontinued, and he was ordered wine, quinine and acetate of lead, with acetic acid and water for drink.

From this date the symptoms progressed in intensity, until the patient gradually sunk; no hæmorrhage having taken place during the whole course of the disease with the exception of the small quantity noticed above. The appearance of the patient three days before death was frightful from the enormous tumefaction of the features. The face was double its natural size, partially covered with several effusions of black blood into the subcutaneous cellular tissue, while the interjacent spaces were of a dark red colour, and the blood in these seemed to be confined to the cutis. The gums were removed by ulceration, laying bare the alveolar processes; sloughs were separating from the inside of cheeks, and although every attention was paid to cleanliness and ventilation, the effluvia emanating from the patient had so impregnated the atmosphere of the apartment as to render it scarcely bearable. The livid spots on the body have become confluent, and those on back are again coalescing with those on the chest and belly. The penis and scrotum are one mass of discolouration. There are no deep ecchymoses on the body similar to those existing on the face. The original petechiæ on the legs have completely disappeared. He expired quite intelligent at 4 o'clock on Wednesday afternoon, the 24th of the same month. Besides the medicines mentioned in the report, the patient, during the course of his treatment, had repeated purgatives with drinks acidulated with the mineral acids —large doses of the hydriodate of potass, and occasional opiates.

*Dissection.*—The relatives of the patient prevented the examination from being as minute as we would have wished. The organs contained in the thorax were all healthy in their texture; on the pericardium and beneath the pulmonic and thoracic pleura, however, there were numerous effusions of black blood, about double the size of the petechiæ occurring in fever. In the abdomen several spots having the same appearance were situated under the peritoneal covering of the parietes, liver, stomach and intestines. As Professor Thomas Thomson had kindly promised to analyze the blood, Mr. Lyon and I were both desirous of obtaining a sufficient quantity to enable him to prosecute the enquiry, but we were much disappointed, after using the utmost care and opening every internal blood-vessel of any consequence, at being able to collect only about two ounces.

I have considered it unnecessary to enter into the details of the case after the few first days, as the practice, being founded on no true pathological knowledge of the disease, was much varied: the symptoms becoming equally aggravated under venesection and the depressing effects of colchicum as under tonics and a liberal allowance of wine. The most remarkable features in this case were the acute rheumatism of the joints—the *pain, heat, redness, and swelling* of the face attending the purpuragic ecchymoses—the small quantity of blood found on inspection, though next to none had escaped from the mucous surfaces—and the great extent of the sanguiferous effusions under the cuticle causing permanent discolouration of more than half of the body. Several of the circular petechiæ on the body, when about the size of a half-crown piece, presented a novel phenomenon in their enlargement. Immediately in contact with their circumference the cuticle was elevated into white prominent rings, having exactly the appearance of the wheals in urticaria, and receding before the extending petechiæ. No fluid, similar to that obtained by Mr. Plumbe from the white protuberances of nettle-rash, exuded when the cuticle of these rings was punctured. This circumstance, together with the pain and heat of the face, plainly indicated a *local* excitement of the cutaneous vessels; but as these symptoms were preceded by the ecchymoses, they must be considered the effects not the causes of the effusions.

Dr. Thomson found that the blood sent him contained all the ingredients of healthy blood, though from the small quantity he was unable to determine whether they existed in the relative proportions of natural blood.

### II. Poisoning by Corrosive Sublimate.

May 6th, 1839, I was suddenly called to visit John Ross, aged 15, dyer, this evening, at 9 o'clock. I found him in bed in a crowded room vomiting, with violent efforts and pain, a frothy viscid mucus mixed with blood. He complained of great thirst, very disagreeable taste in mouth, burning pain and tightness of throat, and deglutition is attended with spasmodic contractions of the œsophagus and of the deep-seated muscles of the neck. The burning pain extends down the gullet into the stomach and bowels, and immediately before and during the act of vomiting, the pain becomes of a tearing nature, and is frequently accompanied with an inclination to purge. Belly is contracted and very tender to the touch. Has slight cramps in his legs. The tongue, gums, and inside of mouth are corrugated, and seem as if they had been partially scalded. Pulse is feeble, rapid, and irregular. Countenance contracted and pale. Skin is covered with clammy moisture.

He says that he has had no food since three o'clock this afternoon—that he was at his usual employment the whole of the day, and left it in perfect health at six o'clock—that he then came home with his companion James Macmillan, a fellow-workman, who invited him to his house, which is situated in the floor above, and there gave him a dram-glassful of liquor which Macmillan stated was whisky—that he drank the greater part of it, but refused to drink it all, as it was too strong and burning, and that immediately after this he became sick and began to vomit. I sent for Macmillan, who unhesitatingly denied that he had given anything to Ross, and repeatedly asserted that the only thing he had in his house was a smoke of tobacco. From the severe agony and the frequent vomitings of the patient interrupting his narrative, nearly an half-hour was lost before I obtained a satisfactory account of the symptoms and the relative circumstances.

I experienced no difficulty in determining that the symptoms the boy Ross presented were induced by a corrosive poison, but the point for my usefulness in treating the emergency was to discover the particular irritant. It could not have been a concentrated mineral acid, because there was neither corrosion nor whitening of the lips or inside of mouth, although upwards of two hours had elapsed since the poison was swallowed. It could not have been oxalic acid, because the patient distinctly declared that the liquor he drank was not acid, but had a very bad bitter taste. Arsenic being the poison most easily obtained was naturally suspected, but Ross stated that Macmillan poured the liquid from a bottle into a glass in his presence, that no sediment fell to the bottom, and that it had the smell of, and was colourless like whiskey. Moveover the early appearance of the symptoms of irritation (within ten minutes of swallowing the liquid) and their excessive violence indicated that the poison must have been a more soluble and a more powerful irritant than arsenic. A review of these circumstances, and a knowledge that the pain and sense of acidity in the throat preceded the derangement of the stomach, led me to conclude that the patient had swallowed a strong solution of corrosive sublimate. Acting on this opinion, I administered a few grains of the sulphate of zinc in a quantity of warm milk which was at hand, and as soon as it could be got, the white of egg. Upon leaving my patient I was followed by Macmillan, who inquired into the cause of Ross's present illness. I informed him that I had no doubt but that he had taken corrosive poison. He then asked me to accompany him to his own house, and see if I could obtain any such poison. I was there shown several bottles, one of which contained a strong spirituous solution of the bichloride of mercury. I searched for the glass (in which Ross had said that he left about a third) and found one containing a liquid, having a taste similar to the contents of the bottle. These I took with me, and delivered to the sheriff.

May 7th. Ross was removed last night to his father's house, where Mr. Hay and I visited him this morning at 5 o'clock. Has had constant vomiting of a bilious fluid containing clots of black blood, and several stools of the same description. The symptoms of inflammation throughout the whole course of the alimentary canal are excessively violent. Features collapsed and slightly bluish —cold sweats—pulse quick and scarcely perceptible. He seemed sinking so rapidly that I felt myself called upon to inform him that I considered him dying. Under this impression he repeated his former statement of the affair. I saw him the same afternoon, when the authorities took his declaration, and he then, in addition to the other symptoms, manifested a strong inclination to doze between each question. He lingered in this collapsed state until the evening of the 9th, when he rallied for a few hours, and mercurial erethysm supervened—the flow of saliva, however, always continued moderate. He afterwards assumed a typhoid appearance, and died comatose on Sabbath morning the 12th, at 5 o'clock, having survived five days and ten hours. During the whole course of his illness he passed no urine.

*Dissection.* The body was inspected thirty hours after death, on a warrant from the sheriff, by Dr. Spittal, in the presence of Dr. Corkindale, Mr. Hay, and myself. No examination was made of the brain. The tongue was swollen, the gums extensively ulcerated and black, and the internal coat of the gullet was much eroded from the mouth to the stomach. Externally the stomach and several parts of the intestines presented a livid appearance, and were double their natural thickness. On opening the stomach, a considerable quantity of a dark grumous fluid escaped, which was exceedingly fetid. The villous coat of this viscus was completely disorganized, and had either sloughed or was hanging in partially detached stripes. In some parts the remaining coats were three-fourths of an inch thick. The mucous membrane of the intestines was generally softened and presented numerous patches of black extravasation. The bladder was much contracted, and of the shape, though not near the size, of the unimpregnated uterus. The other organs were natural. There was no venereal disease. The symptoms during life, and the morbid appearances after death, were quite conclusive as to the cause of death, without analysis of the contents of the stomach, which at the distance of time, and owing to the frequent discharges by vomiting, could not have retained any of the poison in its primary form. I however detected, six months afterwards, in the tissue of the stomach, which had been preserved in spirit of wine, numerous minute white portions of the insoluble compound formed from the antidotes exhibited.

Macmillan, no motive for the commission of the crime being produced against him, was found guilty of culpable homicide at Glasgow in September last. No facts of medical importance were elicited on the trial. The bottle and glass formerly noticed contained a spirituous solution of the bichloride of mercury, in the proportions of one part of the salt to eight of the spirit. The strength of the solution, the quantity taken, and the empty state of the stomach, sufficiently account for the violence of the symptoms, and the collapsed state of the patient at my first visit.

W. REID.

15, Orr Street, Glasgow,
25*th Nov.* 1839.

---

### Some Observations upon the Treatment of Infantile Remittent Fever. By Dr. *M'Cabe*, Hastings.

I have read in the last number of this Journal a " highly graphic" (as it is there justly named) description of the *symptoms* of the above, not unfrequent disease, from the pen or pens of Drs. Bright and Addison. Their description of this complaint cannot be too often placed before the medical public, and therefore (under the hope of indulgence from the Editors) I transcribe the passage.

" When a child from one to eight or ten years of age, has a pale, muddy, or dejected countenance, a foul tongue, a tumid belly, an irregular appetite, and a morbid state of the bowels; when it picks its nose, and is fretful and restless when awake, and whines, moans and starts in its sleep, &c."

I have had, during the last fifteen years at Hastings, a tolerably large practice in complaints of children of the abovenamed ages, and have experienced the most decided good results from under vomiting doses of ipecacuanha, given *twice* a day. Eight or ten days' perseverance in this simple treatment, I have seen followed by results beyond my most sanguine expectations.

Within the last month I was applied to in the case of a boy nine years old, labouring under most of the symptoms above detailed, with, besides, very considerable tumefaction of the glands of the neck; the boy was miserable and unhappy, and made all about him equally so, in a week he was perfectly restored to health, expressed a strong desire to return to his school.

To strengthen the constitution after these attacks, I have seen much benefit from small doses of quinine, or the nitric acid in syrup, twice or thrice a day; a form in which children may be induced to take " doctor's stuff."

---

### TIMBER, CANVASS, CORDAGE, AND WOOLLEN PRESERVATIVE COMPANY.

### SIR WILLIAM BURNETT'S PATENT.

" IT has ever been a great desideratum to all connected with the Maritime Interests of this Country to obtain some means of preserving Timber, Sails, and Cordage, from Dry Rot, Mildew, and the destructive influence of the elements. Sir William Burnett, Physician General of the Navy, whose attention in the course of his public duty has been long drawn to the various, but hitherto ineffectual means resorted to for the preservation of the Ships, Sails, and Cordage of the Royal Navy, has, at length, happily discovered a compound which unites every object sought for by the most sanguine. This discovery he has submitted, as well to the strongest individual and comparative tests by scientific gentlemen, as to the severest trials under his own personal inspection, and the result leaves no doubt of its being adequate to all the desired purposes.

The immense importance of the discovery is too obvious to require being dwelt upon here; but of the Chemical preparation now proposed to be placed before the Public, it may certainly (upon full proof) be stated that it will protect and preserve Timber, Cordage, Canvass, and similar substances from Dry Rot, Mildew, and the elements of decay arising from damp, want of atmospheric circulation, exposure to weather and sea-water; against the influence of the latter of which, it is believed, that no other specific has yet been discovered.

Experiments, the most trying in their nature, have been invariably followed by results the most satisfactory. and conclusive. Specimens of prepared and unprepared Timber, and also of Canvass, Cordage, and Woollen Cloth have been subjected to the severe tests of the Fungus Pit, at Woolwich Dock Yard, and of other suitable places, for various periods, under official superintendence; and, upon after-inspection, the *prepared* were found unaffected, whilst the *unprepared* were in a state of decay.

Experiments have also been repeatedly made upon Copper and Iron Bolts with the most satisfactory results.

But the trials which Cordage, Canvass, and Woollen Cloth, prepared with this Compound have undergone, do most incontestibly prove the superiority of this process over any other in use, and establish the fact, that Salt-water so far from hastening the decay of articles prepared under this process, or from neutralizing its effects, has, on the contrary, the quality of increasing its efficacy ; a property, which cannot fail to ensure encouragement commensurate with its vast importance to the Maritime Interests of the Country! whilst its general adaptation for use Ashore and Afloat must command the patronage both of the Landed and Maritime Interests, and ultimately lead to universal adoption.

This process is moreover perfectly innoxious, and cannot by possibility endanger health, either in its preparation, or in its application: All the Timbers and Ceiling of a Ship may, therefore, be impregnated with the solution without the slightest prejudicial effect to the crowded inmates of its close confines.

It purifies Bilge-water.

It is not only free from any quality calculated to promote Oxydation of Metals, but actually retards this process.

And further, this valuable preparation is *comparatively unexpensive in its use.*

In consequence of these favourable results, Sir William Burnett has been induced to take out a Patent for this his Invention; but his official duties rendering him unable, singly, to devote the time and attention requisite to bring his important discovery into operation, it has been proposed to form a Company for the purpose of carrying that object into effect."

Through the kindness of Sir William Burnett, we have had an opportunity of seeing the various specimens of wood, cordage, canvass, and woollen cloths imbued with the solution of chloride of zinc, (being the basis of Sir William's patent,) as compared with similar specimens unembued with the solution, and all equally exposed to the action of wind, weather, damp, and the causes of dry rot. The freshness, tenacity, and strength of the imbued materials, presented a remarkable contrast with those qualities—(or rather absence of those qualities)—in the same kind of materials prepared with the solution. The complete extinction of the abominable smell of bilge-water (sulphuretted hydrogen) by mixture with a few drops of the solution, was most striking. Upon the whole, we augur well of this discovery, especially as the agent is totally devoid of any noxious quality.

---

### Dr. Lynn's Case.

*To the Editor of the Medico-Chirurgical Review..*

Dispensary, Market Street, Aug, 15, 1839.

Dear Sir,—You will oblige me by inserting the following Case in the next Number of your excellent Journal.

I am, faithfully yours,
J. M. Lynn, M.D.

In the beginning of January, 1838, I was called upon to attend Mrs. S——, in her first labour. On arrival, I found that she was a person of strong muscular fibre, and somewhat advanced in life. On examination the parts felt remarkably rigid, and although the pains were very sharp, and had been increasing in severity for the preceding three hours, very little progress had been made.

After a very severe and tedious labour, she was delivered by the efforts of Nature of a female child, whose head was greatly deformed by the long continued pressure, so much so, that the lateral halves of the os frontis overlapped each other in the mesial line from the nose to the anterior fontanelle.

I was unable to replace the bones, and I knew of no author from whom to seek for advice upon the subject.

The poor infant soon shewed symptoms of cerebral disturbance, which has since degenerated into hopeless idiocy.

My object in requesting for this case a corner in your highly practical Journal, is to draw professional attention to this deformity, as a frequent cause of idiocy, and hoping that some plan might be suggested for removing this congenital malformation.

I may add, that I have since had an opportunity of examining the heads of two other idiotic infants, in both of whom I found the same osseous displacement.

---

### OPERATION OE SCARIFYING THE CERVIX UTERI.

*To the Editor of the Medico-Chirurgical Review.*

Sir,—I am anxious to communicate the following operation, as I have reason to believe that superficial scarification of the cervix uteri, in inflammatory congestion of the uterus, accompanied by very severe and painful dysmenorrhœa, is nearly, if not entirely, an original suggestion—especially with regard to abstracting from it a definite quantity of blood. Dr. Ashwell, of Guy's Hospital, who saw the case with me, evinced his usual benevolent interest therein, was much pleased with the effect of the operation, and requested that it might be repeated as circumstances required. I remain, Sir, yours, respectfully,

J. L. FENNER.

15, King's Row, Pentonville,
Jan. 18th, 1840.

Mrs. ———, a widow, æt. 39, had been long afflicted with dysmenorrhœa, accompanied with inflammatory congestion of the uterus, dating its origin many years since, from a severe and protracted labour. The nervous system was so entirely implicated in this affection, that the superior and inferior extremities, as well as the body, were continually agitated by a species of chorea. She was passing through a three months' course of mercurial friction, and had found no relief from opium or any kind of narcotics. Leeches alone, applied round the *cervix* uteri, with my speculum, had palliated her sufferings, and these acted like enchantment, dissipating every symptom, and, after restless nights, producing a calm, refreshing sleep of some hours' duration. Appreciating the relief obtained from the abstraction of blood, and its tendency to remove congestion, it struck me as quite practicable, aided by my cylindrical tubular speculum (described in The Lancet, May 18, 1839, and to be seen at instrument makers,) easily to abstract, by slight scarifications of the cervix uteri, any quantity I might think desirable.

Nov. 1, 1839. After a few superficial scarifications the blood trickled freely, and, in a quarter of an hour, two ounces and a half (by weight) were obtained, and the tube withdrawn, when the bleeding immediately ceased. Precisely the same relief followed, with uninterrupted sleep, as was wont to result from the application of leeches. The patient said that the operation was so *painless* that it would not even have disturbed her sleep!

2. Two ounces and a half of blood were obtained under the same circumstances.

3. Three ounces and a half of blood.

5. The cervix uteri having many marks of scarification, the tube was withdrawn a little, so as to expose the *cul de sac* of the vagina. Scarifications were made, presuming that it would bleed freely, because to that part of the vagina leeches have been applied by tubes perforated at the end with holes, and unscientifically thrust up the vagina; but by such tubes leeches cannot be duly applied to the *cervix* uteri, though they may sometimes to a portion of its side. The blood trickled freely, and in a quarter of an hour four ounces (by weight) were obtained, with the same relief as by leeches.

9. The patient having obtained more decided relief than on any former occasion from the application of leeches, the scarification is to be resumed as occasion may require, and the mercurial friction to be continued to the given time. The distressing soreness and ulceration of the mouth and gums from mercury are prevented, and the patient enabled to eat comfortably, by the constant use of Dr. Darling's excellent preparation of chloride of soda, obtained from Mr. Garden.

Having received numerous inquiries respecting the best mode of using my speculum, and also relative to the steps of the operation of scarification of the cervix uteri, I take this opportunity of explaining. The proper position of the patient is to recline on the back, the feet resting on the edge of the bed, I have no intention of entering into the controversy on the use or the abuse of the speculum; but I wish to say, that in my own practice, and when properly conducted, there is very little exposure. A metallic tube, of one of my three sizes, adapted to the individual case (1 inch, 1¼, 1½,) with the corresponding boxwood cylinder, *fig.* 2, fitted to the wooden handle, and introduced to the end of the tube, until it stops, is to be lubricated with some unctuous substance. The introduction of my speculum is always to be accomplished without producing any positive pain. Pressure is to be made with a gentle, semi-rotatory motion, entirely on the wooden handle, held by the thumb and two fingers of the right hand, and in the exact direction where the os uteri has previously been ascertained, by the taxis, to be situated; the thumb and index finger of the left hand, *fig*. 1, are applied to hold the tube. The box-cylinder having passed the perineal portion of the vagina, and entered the pelvic cavity, the resistance has ceased; and the wooden handle attached to the cylinder being withdrawn, the most perfect view of the os and cervix uteri is obtained, corresponding with the diameter of the tube, as in *fig*. 5, either by the natural light or that of a taper.

The tube is made of the same diameter throughout, to obtain the largest possible view, and the effect of the conical box-wood cylinder accurately fitting the tube, produces previous dilatation before the tube (its edges being thus protected by the cylinder) comes in contact with the vagina. If a minute or two are occupied in preparatory dilatation towards the perineum, a larger sized tube than otherwise may be introduced, always, it is to be understood, without producing positive pain. Thus, with the aid of the cylinder, I now use the inch-and-a-half diameter, where formerly I should not have attempted doing so; and thereby I obtain a more important view. The elastic strap of narrow Indian-rubber, wet, *fig*. 1, then securely fixes the speculum, by the small hook being attached to the tube, and the large hook to the patient's drawers or clothes. All other specula employ one hand to hold them in their situation. Thus both hands of the operator are at perfect liberty for the operation of scarifying the cervix uteri, or the usual ones connected with the injection of the uterus, the application of caustic to abrasions or ulcerations of the os and cervix, leeches around the cervix, &c. The speculum is, therefore, indispensably necessary, not merely because the unassisted sense of touch is insufficient to discover the morbid phenomena thus disclosed to the sense of sight, but is equally so as a medium for the local application of various medicaments. The state of the whole extent of the mucous coat of the vagina may be most minutely explored by slowly withdrawing the tube. The accompanying sketch illustrates the steps of the operation of scarification of the cervix uteri. The speculum being duly introduced to expose the cervix, and secured by the elastic Indian-rubber strap, *fig*. 1. The little mop, *fig*. 4, made by tying lint or wadding on a skewer of wood six inches long, is first used to remove mucus, and enable the structure to be clearly examined. A lancet, mounted on a similar stick, is held by the thumb and two fingers of the right hand, and used like a pencil, making superficial scarifications, transversely, from $\frac{1}{16}$th to the $\frac{1}{8}$th of an inch in depth. These transverse scarifications must commence from below, that the subsequent lines may not be obscured by blood. *Fig*. 5 shews the operation thus completed, which will furnish about three or four ounces of blood. But where the abstraction of a greater quantity is desirable, the incisions are crossed perpendicularly to the first, as seen in *fig*. 6, which, in a few minutes, will produce six or eight ounces of blood. The tube being kept in a depending position, the blood trickles freely through it into a saucer placed underneath; and to prevent obstruction to its flow, I remove coagula by means of a scraper made of a bit of bonnet-wire, tied to a stick, and bent, as represented in *fig*. 3.

In my own practice, this operation will supersede leeches, which on many accounts, are objectionable to both parties; in the one, exciting a degree of alarm and anxiety, and, in the other, taxing to the utmost the virtue of patience. When leeches are used, I have found that they live longer, and are rendered more useful, by putting them in the gorged state into tepid water, and keeping them in a warm situation. Practitioners will find, that a greater degree of relief is almost instantly obtained.

---

### Some Notices of recent German Publications.

We are indebted to a kind and very able friend for the following. It arrived too late for insertion in the Review Department, and we are compelled to introduce it here. Two notices, for which we have not space at present, will be found in our next number.

Die Bewegung der Crystallinse, von Prof. Dr. A. *Hück*. Dorfat, 1839.

### The Motions of the Crystalline Lens, &c.

The author in this paper treats of the nature of short and long sight, founding his observations upon a series of experiments made during the course of thirteen years. He first adverts to the well known fact, that when the eye perceives a distant object distinctly, a nearer object becomes indistinct, and vice versâ. Distinct vision is limited; a healthy eye cannot see distinctly nearer than about five inches; this point the author calls the limit of distinct vision, and the healthy eye can see distinctly to the greatest distance beyond it. (Chapter 2.) The short-sighted eye can only see distinctly to a certain distance, which the author designates the far-point: between the limit-point and far-point, the short-sighted eye sees distinctly, but neither nearer than the former, nor farther than the latter. The far-sighted eye sees, on the contrary, at a distance, as distinctly as the healthy eye, only the limit-point is farther from the eye. The concurrence of both diseases is rare, but was observed in a physician who could not see distinctly nearer than fifteen inches or farther than twenty-eight. It still more rarely occurs that vision is distinct at one distance only, and indistinct at a distance either more or less remote. Farther on (chapter 3,) the author distinguishes between distinct vision and acute sight, and demonstrates by two hundred experiments on different individuals, that the acute-sighted eye sees thus acutely both at near and remote distances; so that this kind of sight does not depend upon the refracting media, but upon the sensibility of the retina. Objects disappear under the same angle of vision,—a small object, for example, at the distance of eleven inches, a larger object at the distance of three hundred feet. (chapter 4.)

There is not for a healthy,—a long,—or a short-sighted eye, one special distance of the most distinct vision, (Heroptu,) but it changes with each act of vision. (chapter 5.) By means of other experiments upon his own vision, with the eyes of animals and with glass lenses, the author explains the nature of indistinct vision, double sight through two small openings, and distinct sight through one, &c. (chapter 7.) Many phenomena of vision depending upon internal changes in the eye, as well as those observed in the short-sighted eye, are explained upon these principles. The author here takes occasion to remark that most physiologists who have treated this subject as Iscoiranus, Purkinge,

&c. were themselves short-sighted, a circumstance which led them to false conclusions.

The 2nd section treats of the probable causes of the internal changes. The 8th chapter contains a critique of the different views of various authors on this subject, viz. Simonoff, Littleton, Majendie, Serres, Jurin, Crampton. In the 9th chapter it is shewn that a change in the form of the eyeball, supposed to be induced by its mucles, is impossible, and especially that the experiment of Home is unsatisfactory. In the appendix the author remarks that it has been distinctly shewn by the recent experiments of Professor Senff that the convexity of the cornea does not change in near and remote vision. The author further remarks that the situation of the muscles is opposed to the idea of compression, and that lateral compression of the eye in a living animal is insufficient to render the figure of the image of an internal object more distinct upon the sclerotica.

Finally, the experiment of the application of henbane demonstrates the complete independence of the internal changes in the eye, of muscular action. In the 10th chapter it is shewn that dilatation of the pupil has no connexion with the other internal changes. The author principally endeavours to combat the opinion of Treviranus, and shews that his calculation is not based upon certain data, and that its consequences do not coincide with experience. In repeating the experiment with the extract of hyosciamus, the author found, what had escaped previous observers, that the pupil dilated before the power of adaptation was weakened, and contracted before the restoration of that power took place. Chapter 11 explains the impossibility of change in the form of the lens by the contraction of any part of its structure.

The 3rd section proceeds to point out a motion and a change in the convexity of the crystalline lens, as the cause of the internal changes. The author notices (chap. 12,) a convexity of the iris, as occurring in several persons during the vision of a near object. He observed the same in birds and in mammalia. That this convexity of the iris was caused by the lens, was distinctly and incontrovertibly proved after the death of these animals.

When the lens was moved forwards, in the eyes of kittens, by means of a needle introduced at the side, and moved in a lever-like manner, the image on the sclerotica of a near object was rendered more distinct. After giving the different opinions upon the mode in which the lens is moved and changed in form, (chapter 13,) he enters upon an accurate and anatomical examination of the corpus ciliare and the surrounding parts, which he illustrates by drawings. The result of his researches upon the human eye, and upon the eye of 24 different species of mammalia, but especially of the lynx and the dog, is, that the folds of the corpus ciliare form cavities which are filled with the fluid and are in connexion with the flat chief canal of Petit, and lie in such a manner between the borders of the ciliary processes, as to form a circular roll round the border of the capsule of the lens. The corpus ciliare of birds is described in 12 different species, but for the anatomical details we must refer to the work itself. In the 17th and concluding chapter the influence of the corpus ciliare upon the lens, is described in man and the mammalia, and in the appendix Professor Senff, relying upon the admeasurements given by Kraus, and the refractive powers of the eye given by Brewster, shews that the mere advancement of the lens forward, even to the cornea, would be insufficient to explain near sight, whilst a lateral compression which shortens the diameter of the lens only an eighth, brings the eye into a state to admit of distinct vision at the distance of about five inches.

## Figures and Tables

**Figure f1:**
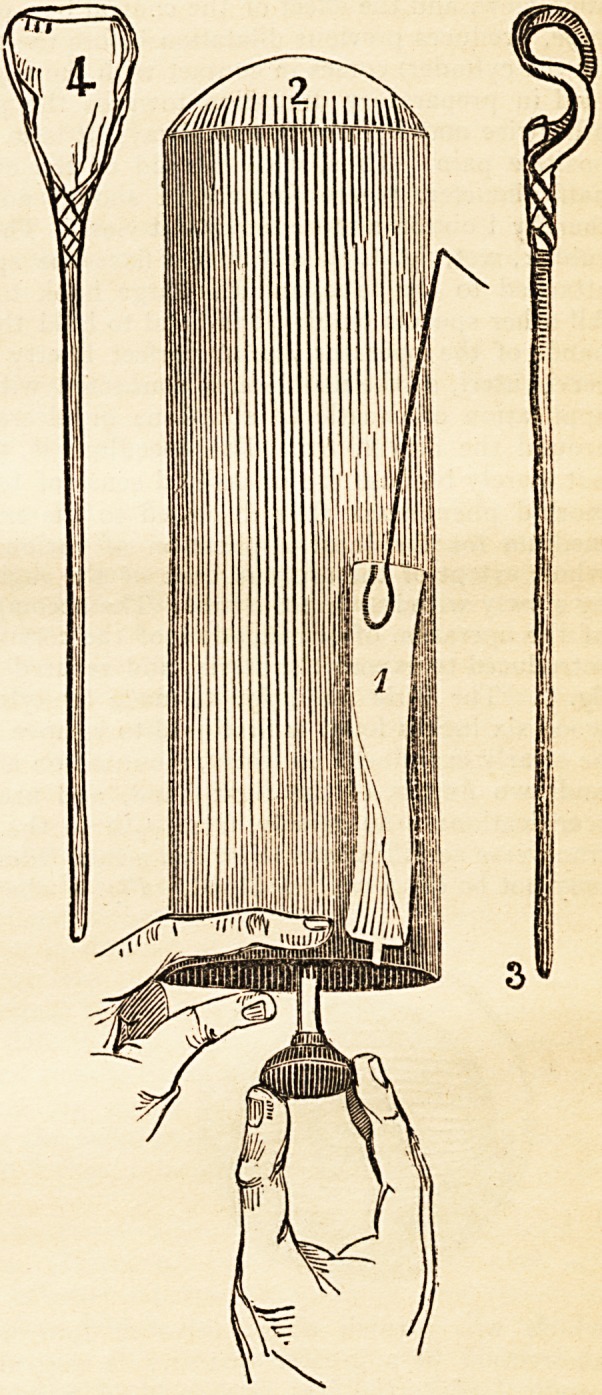


**Figure f2:**
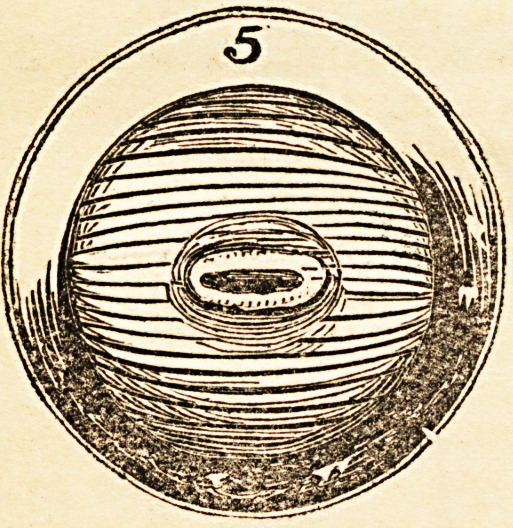


**Figure f3:**